# Molecular Screening and Field Validation of Potato Germplasm for Resistance to Potato Cyst Nematodes

**DOI:** 10.3390/plants15172686

**Published:** 2026-09-01

**Authors:** Xi Yang, Zongming Guo, Yanchao Yue, Baoyan Song, Huachun Guo

**Affiliations:** College of Agronomy and Biotechnology, Yunnan Agricultural University, Kunming 650201, China; yangxitangrun@outlook.com (X.Y.); darkskysaber@outlook.com (Z.G.); yyc000409@163.com (Y.Y.); 19187689949@163.com (B.S.)

**Keywords:** potato, cyst nematodes, field resistance, molecular screening, germplasm resources

## Abstract

Potato cyst nematode (PCN; *Globodera* spp.) is an internationally quarantined pest threatening potato production in Yunnan, Sichuan, and Guizhou, China. We screened 418 potato accessions using markers 57R (*H1*), Gro1-4(*Gro1-4*), and Gpa2-2(*Gpa2*). Detection frequencies were 40.43% (Gpa2-2), 14.35% (57R), and 3.11% (Gro1-4); 50.48% carried at least one marker, 7.42% carried two markers, and none carried all three. Based on marker genotypes, nine representative accessions (five *H1*-carrying, two *Gpa2*-only, two marker-free) were field-tested in a nursery infested with *Globodera rostochiensis*. All five *H1*-carrying accessions showed no cyst formation (RF = 0), indicating high resistance. The *Gpa2*-only accessions had RF values of 0.37 and 0.58, indicating partial resistance, although *Gpa2* mainly targets *Globodera pallida*. Marker-free accessions were susceptible (RF ≥ 0.79). According to international criteria (RF < 0.1 considered highly resistant), five accessions—‘Dianshu 1208’, ‘Dianshu 14023’, ‘Dianshu 1520’, ‘Diancaishu 102’, and ‘Atlantic’—were confirmed to be highly resistant. These results indicate that *H1* is associated with effective resistance to *G. rostochiensis* in the tested accessions and highlight the value of combining marker-assisted selection with field validation for reliable PCN resistance identification.

## 1. Introduction

Potato (*Solanum tuberosum* L.) is an herbaceous plant in the family Solanaceae, genus Solanum [1]. It ranks as the third largest food crop globally, with tubers rich in starch, amino acids, vitamins, anthocyanins, and other nutrients [2]. Owing to its high nutritional value and broad adaptability, potato is cultivated extensively worldwide. Although China ranks as the world’s largest potato producer in terms of both harvested area and total output, per-unit yield remains below the global average. Consequently, improving yield and quality has become a primary objective for advancing China’s potato industry [3].

Among the biotic constraints on potato production, potato cyst nematode (PCN) is recognized as one of the most destructive pests in potato cultivation [4]. Native to the Andean region of South America, this nematode shares a long co-evolutionary history with its host plant, potato [5]. As potato cultivation spread globally, PCN was inadvertently introduced to numerous countries worldwide. The second-stage juvenile (J2) is the primary infective stage, penetrating and establishing feeding sites within potato root systems [6]. By depleting plant nutrients, the nematodes cause stunted growth, chlorosis, and wilting of above-ground tissues, ultimately resulting in substantial reductions in both total and marketable tuber yield [7]. Moreover, once established in a field, the cysts remain viable in the soil for more than 10 years and are extremely difficult to eradicate [8,9]. Consequently, infested production areas are officially designated as quarantine zones, within which both seed potato production and commercial potato distribution are strictly regulated, inflicting considerable economic losses on the local agricultural sector. PCN outbreaks were detected in China in 2022, specifically in Zhaotong (Yunnan), Bijie (Guizhou), and Liangshan (Sichuan) [10]. The Wumeng Mountain Region, which encompasses these three locations, is the largest potato-producing area in China, with an annual sown area of approximately 15 million mu (ca. 1.0 × 10^6^ ha), accounting for one-sixth of the national total. This region is also among the 14 contiguous impoverished areas designated by the Chinese government; PCN introduction therefore poses a direct threat to the local potato industry and to the livelihoods of farming households [10].

Current management strategies for PCN primarily rely on the application of chemical nematicides, crop rotation, and the use of resistant cultivars. However, the use of chemical nematicides has raised concerns due to their environmental persistence, non-target toxicity, and the potential development of resistance in nematode populations. As a more sustainable alternative, biological control has attracted increasing attention. Edible mushroom bioproducts have shown promising nematicidal activity against both plant- and animal-parasitic nematodes [11,12]. Moreover, recent advances in plant nematology have summarized various biological control strategies, including the use of bacteria and nematophagous fungi [13]. Despite these advances, the integration of biological control with host resistance has not been extensively explored, and the combined effects of resistant cultivars and biocontrol agents against PCN remain poorly understood. Therefore, the identification and deployment of effective resistance genes remains a priority for sustainable PCN management. Research on PCN resistance began internationally in the mid- to late twentieth century. Through systematic screening of resistant germplasm, several key resistance genes have been identified in cultivated potato varieties and their wild relatives [14]. Resistance genes cloned or mapped against the golden potato cyst nematode (*Globodera rostochiensis*) include *H1* [15], *Gro1-4*, *Gro1*, *GroV1*, *Ro2*, and *Hero* [16,17], while those conferring resistance to the white potato cyst nematode (*Globodera pallida*) include *Gpa2* [18], *GpaM*, and *GpaV* [19]. Among these, *H1*, derived from the Andean cultivar (*Solanum tuberosum* ssp. *andigena*), maps to the telomeric region of chromosome 5 and confers high resistance to the Ro1 and Ro4 pathotypes of *G. rostochiensis* [20]. *Gro1-4*, originating from the wild species *S. spegazzinii*, is located on chromosome 7 and reportedly confers resistance to all known physiological races of *G. rostochiensis* [21]. *Gpa2*, also derived from an Andean cultivated variety, maps to chromosome 12 and exhibits high resistance to *G. pallida* race Pa2/3 [22]. These three genes, characterized by their broad-spectrum and robust resistance, have become core genetic resources in contemporary breeding programs. Several gene-linked markers—including TG689, 57R, N146, Gro1-4, Gro1-4-1, and Gpa2-2—have been developed for use in marker-assisted selection. Markers tightly linked to *H1* include TG689, 57R, N146, and N195 [23]; those linked to *Gro1-4* include Gro1-4, Gro1-4-1; and those associated with *Gpa2* include Gpa2-2, 77R, and GP34 [24,25]. In recent years, 57R has gradually superseded earlier markers owing to its superior reliability, and has become the preferred marker for *H1* genotyping [26].

In China, breeding for PCN-resistant potato varieties commenced relatively late, although molecular screening of resistance genes has been increasingly conducted in recent years [27,28]. Preliminary findings indicate that the frequency of PCN resistance genes in domestic germplasm remains generally low. Nevertheless, two important gaps persist in the current literature: (i) the correlation between molecular marker genotypes and field resistance phenotypes has not been systematically validated in Chinese germplasm; and (ii) most studies rely solely on marker data without corroborating evidence from field-based resistance assays. In response to the 2022 PCN outbreaks in southwestern China, this study aimed to determine the prevalence of the *H1*, *Gpa2*, and *Gro1-4* resistance markers in 418 potato accessions from Chinese and CIP-derived germplasm, evaluate their correlation with field resistance under local pathotype conditions, and identify resistant parental lines for regional breeding programs.

## 2. Results

### 2.1. Overall Distribution of Resistance Genes

**Figure 1:**
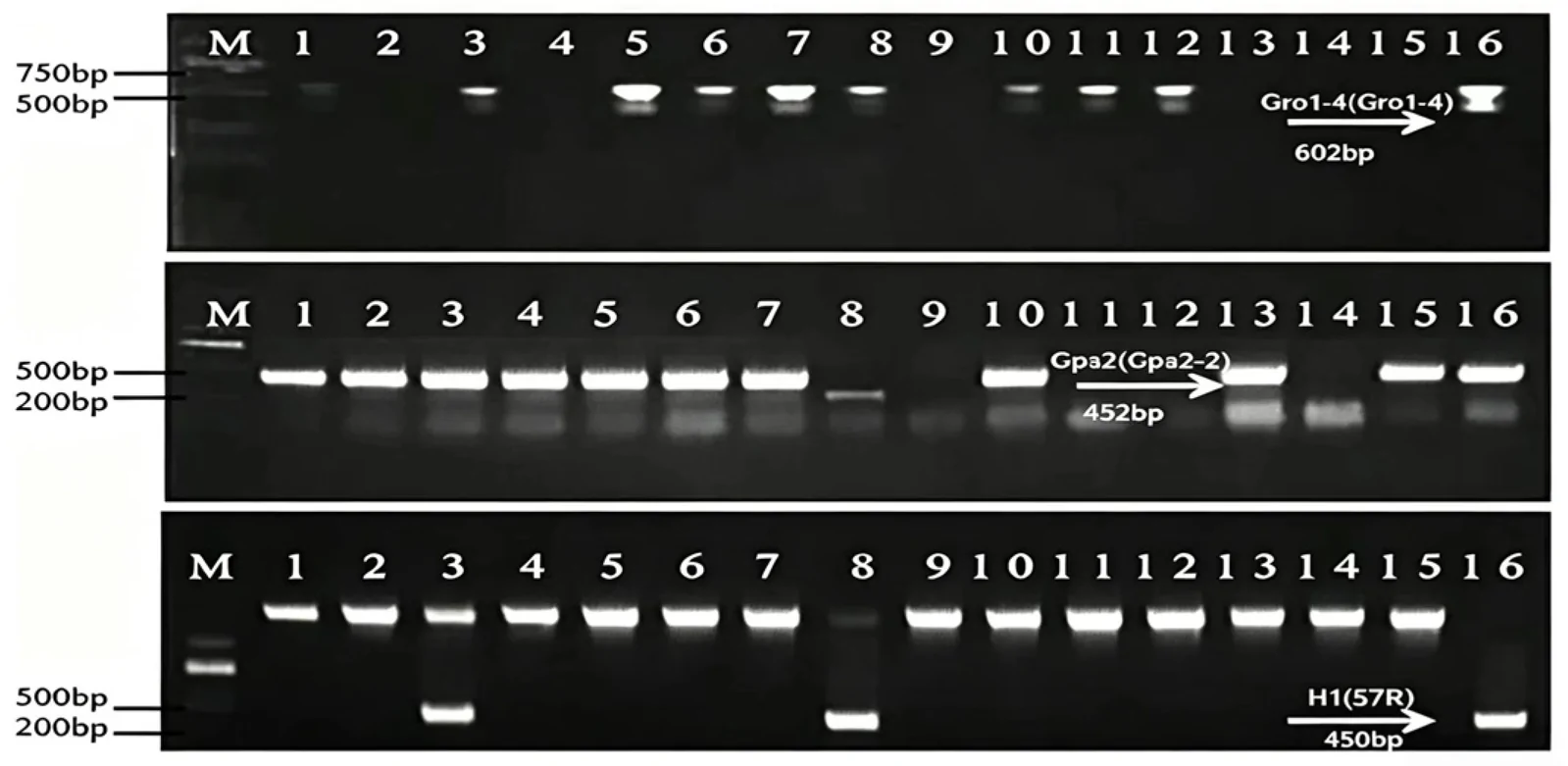
PCR detection of three resistance gene markers in potato germplasm. M: BM 2000+ DNA Marker. Lanes 1–16: *Gro1-4* gene detection (Gro1-4 marker). 1: 399049.14; 2: 399049.16; 3: 399049.22; 4: 399053.11; 5: 399053.15; 6: 399062.118; 7: 399067.14; 8: 399067.22; 9: 399072.11; 10: 399072.28; 11: 399073.23; 12: 399075.32; 13: 399075.7; 14: 399078.11; 15: 399079.22; 16: 399083.4. Lanes 1–16: *Gpa2* gene detection (Gpa2-2 marker). 1: 302498.70; 2: 303381.106; 3: 303381.30; 4: 304347.6; 5: 304349.8; 6: 304350.18; 7: 304350.78; 8: 304351.109; 9: 304351.31; 10: 304366.46; 11: 304368.22; 12: 304371.20; 13: 304371.58; 14: 304387.17; 15: 304387.92; 16: 304394.56. Lanes 1–16: *H1* gene detection (57R marker). 1: 300055.32; 2: 300063.9; 3: 300065.4; 4: 300135.14; 5: 300135.3; 6: 300137.31; 7: 301623.15; 8: 301624.95; 9: 301026.23; 10: 301037.85; 11: 301041.26; 12: 301044.36; 13: 301045.74; 14: 301055.53; 15: 301056.54; 16: 302498.70. The original, uncropped gel images are provided in Supplementary Figure S1.

The markers 57R, Gro1-4, and Gpa2-2 were used to detect accessions carrying the *H1*, *Gro1-4*, and *Gpa2* resistance genes, with the *Gpa2* gene conferring resistance to *G. pallida*. Using these three molecular markers, a total of 418 potato germplasm accessions were screened for PCN resistance (Appendix A Table A2). Accordingly, specific fragments of 450 bp, 602 bp, and 452 bp were amplified from accessions carrying the *H1*, *Gro1-4*, and *Gpa2* genes, respectively (Figure 1).

Among the three markers tested, Gpa2-2, linked to the resistance gene *Gpa2*, exhibited the highest detection rate, with positive amplification observed in 169 accessions (40.43%; Table 1). The marker 57R, associated with the *H1* gene, ranked second, with 60 positive accessions (14.35%). The *Gro1-4* marker showed the lowest detection rate, with only 13 positive accessions (3.11%), all of which originated from CIP, indicating that the *Gro1-4* gene is relatively limited in distribution within this population. Although *H1* is an important source of resistance to *G. rostochiensis*, its detection rate was notably lower than that of *Gpa2* in the present study. The low occurrence of *Gro1-4* may be attributed to its wild origin (*S. spegazzinii*); introgression of this gene into cultivated backgrounds often entails linkage drag or adaptive penalties, which likely limits its retention in conventional breeding germplasm.

The overall distribution of marker combinations (Table 2) revealed that, among the 418 accessions screened, 211 (50.48%) carried at least one of the three markers, of which 31 (7.42%) carried two markers, while none carried all three. The remaining 207 accessions (49.52%) tested negative for all three markers.

The stacking of multiple resistance genes remains quite limited in the current germplasm pool, and the overall frequency of PCN resistance genes—particularly *H1* and *Gro1-4*—is relatively low, highlighting the urgent need to systematically introduce and pyramid diverse resistance alleles in future breeding efforts.

### 2.2. Distribution of Resistance Genes Among Accessions of Different Origins

As described in Section 4.1, the 418 accessions were divided into four groups based on provenance for comparative analysis of resistance gene frequencies. Based on their provenance, the 418 accessions were categorized into four groups: CIP introductions (251 accessions), Yunnan landraces (10 accessions), breeding lines (118 accessions), and other Chinese cultivars (39 accessions). Differences in resistance gene distribution among the four groups were further compared (Table 3).

Chi-square tests were performed to compare the frequencies of each resistance marker among the four origin groups. For *H1*, no significant difference was detected (χ^2^ = 0.719, df = 3, *p* = 0.869), indicating a relatively even distribution, with detection rates ranging from 11.86% to 20.00%. By contrast, a highly significant difference was detected for *Gpa2* (χ^2^ = 83.826, df = 3, *p* < 0.05), with detection rates highest in CIP introductions (58.17%), followed by breeding lines (16.95%), Yunnan landraces (10.00%), and other Chinese cultivars (5.13%). For *Gro1-4*, because more than 20% of cells had expected frequencies below 5, Fisher’s exact test was applied instead of the chi-square test, and the result likewise revealed a significant association with origin (*p* = 0.028). Notably, all 13 positive accessions were confined to CIP introductions, whereas none were found in domestic materials, underscoring the extreme rarity of this gene in Chinese germplasm.

### 2.3. Field Resistance Testing Results

Significant differences in cyst counts were observed among the nine tested varieties (F = 60.912, df = 8, 18, *p* < 0.05, one-way ANOVA; Table 4). Since Levene’s test indicated unequal variances among groups (F = 8.981, *p* < 0.05), Tamhane’s T2 post hoc test was used for multiple comparisons. The five HR varieties (‘Dianshu 1208’, ‘Dianshu 14023’, ‘Dianshu 1520’, ‘Diancaishu 102’, and ‘Atlantic’) exhibited no cyst formation across all replicates (0 ± 0) and were classified as highly resistant (group a). ‘5S-30’ (15.7 ± 2.1) and ‘Dianshu 1209’ (24.7 ± 4.0) formed a statistically homogeneous group (b), as no significant difference was detected between them (Tamhane’s T2, *p* = 0.266); both differed significantly, however, from the HR group (*p* < 0.05). Based on the RF threshold, ‘5S-30’ (RF = 0.37) was classified as moderately resistant, whereas ‘Dianshu 1209’ (RF = 0.58) was classified as susceptible. Although these two genotypes belonged to the same statistical subset (group b), their RF values fell on opposite sides of the resistance grading threshold (RF = 0.5), resulting in divergent phenotypic classifications and suggesting that statistical clustering does not strictly coincide with discrete trait-based grading criteria. ‘Dianshu 1418’ (33.3 ± 8.39) and ‘Qingshu 9’ (42.3 ± 5.86) were assigned to groups c and d, respectively, each differing significantly from all other groups (*p* < 0.05). The susceptible control ‘Qingshu 9’ exhibited the highest cyst count (RF = 1.00), confirming the reliability of the experimental system.

**Figure 2:**
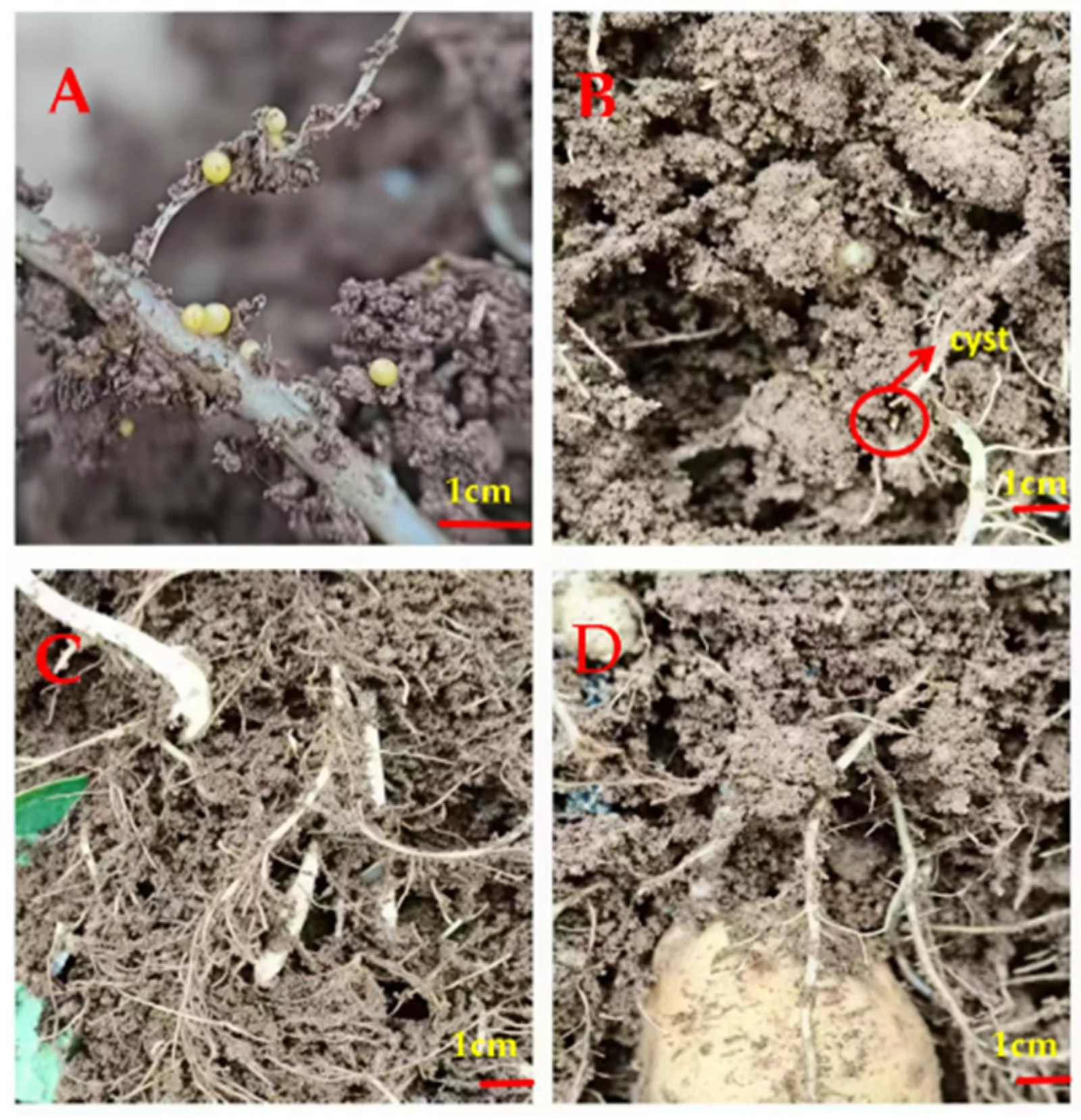
Root cyst attachment of potato varieties/lines with different resistance gene combinations. (**A**) Susceptible control ‘Qingshu 9’ (no marker, RF = 1.00); (**B**) ‘Dianshu 1209’ (*Gpa2* only, RF = 0.58); (**C**) ‘Dianshu 1208’ (*H1* only, RF = 0); (**D**) ‘Atlantic’ (*H1* + *Gpa2*, RF = 0).

Root cyst attachment differed significantly among genotypically distinct varieties (Figure 2A,D). The roots of the susceptible control ‘Qingshu 9’ were heavily infested with pale yellow cysts that aggregated on the fibrous and lateral root surfaces; in some cases, the roots were almost entirely enveloped by cysts. The roots of ‘Dianshu 1209’ (carrying only the *Gpa2*-associated marker Gpa2-2) still harbored a substantial number of cysts, albeit slightly fewer than the susceptible control. Nevertheless, it was rated as susceptible, suggesting that the presence of *Gpa2* alone confers only limited field resistance. By contrast, ‘Dianshu 1208’ (carrying only the *H1*-associated marker 57R) displayed negligible cyst attachment on its root system, with zero cyst counts across all three replicates, confirming a highly resistant phenotype. This observation provides strong evidence for the effectiveness of the *H1* gene against the Ro1 pathotype of *G. rostochiensis*. Furthermore, its root system remained robust, with well-developed and vigorously growing fibrous roots. Similarly, the gene-pyramided variety ‘Atlantic’, carrying both *H1* and *Gpa2* (57R and Gpa2-2), also exhibited clean root systems free of cyst attachment, consistent with the performance of ‘Dianshu 1208’. In theory, varieties pyramided with both genes have the potential to confer resistance against golden potato cyst nematode (*G. rostochiensis*) and white potato cyst nematode (*G. pallida*) simultaneously, and thus hold greater promise for application in complex epidemic areas where multiple PCN pathotypes coexist. These findings demonstrate that the *H1* gene, whether present singly or pyramided with *Gpa2*, provides complete resistance to *G. rostochiensis* under the conditions tested, and thus represents a valuable genetic resource for breeding programs in PCN-infested regions of southwestern China. In contrast, *Gpa2* alone did not provide effective resistance against the *G. rostochiensis* pathotype prevalent in this field, consistent with its known specificity against *G. pallida*. Therefore, in regions where *G. rostochiensis* is the dominant species, incorporation of *H1* remains essential, whereas gene pyramiding with *Gpa2* would be more beneficial in areas where both species coexist.

The field survey results for disease symptoms are summarized in Table 5. Kruskal–Wallis tests detected highly significant differences among genotype groups for both yellowing (H = 16.474, df = 4, *p* = 0.002) and wilting (H = 15.713, df = 4, *p* = 0.003). For yellowing severity, the three *H1*-only varieties and the marker-free ‘Dianshu 1418’ all received a score of 0 (mean rank = 11.50), whereas the susceptible control ‘Qingshu 9’ scored 1 (mean rank = 24.50); the *Gpa2*-only group exhibited an intermediate mean rank of 16.25. For wilting severity, the *H1*-only and pyramided (*H1* + *Gpa2*) varieties showed the lowest mean rank (10.00), followed by the *Gpa2*-only group (16.50), while the marker-free ‘Dianshu 1418’ recorded the highest mean rank (23.00), even exceeding that of the susceptible control (20.00). Notably, ‘Dianshu 1418’ displayed contrasting responses across the two symptoms, scoring 0 for yellowing yet showing the highest wilting severity, suggesting a phenotypic decoupling in this genotype. Collectively, the symptom scores were consistent with the cyst count data, confirming that the *H1* gene confers a dominant and stable effect on reducing above-ground disease symptoms. The DSI values were consistent with the symptom scores: no symptoms were recorded for the *H1*-carrying and pyramided genotypes (DSI = 0%), whereas the *Gpa2*-only and marker-free accessions exhibited mild-to-moderate symptoms (DSI ranging from 16.7% to 50%), and the susceptible control ‘Qingshu 9’ showed the highest disease severity (DSI up to 83.3%).

## 3. Discussion

In the present study, screening of 418 potato accessions revealed that *Gpa2* had the highest detection rate (40.43%), followed by *H1* (14.35%), while *Gro1-4* had the lowest (3.11%). This differential distribution pattern likely reflects the varied introduction pathways and temporal dynamics of resistance genes over the course of potato breeding in China. The relatively high frequency of the *Gpa2* gene observed in this study may be attributed to the extensive use of its donor parent, the Andean cultivar *Solanum tuberosum* ssp. *andigena* CPC 1673, in CIP’s potato breeding pipelines [29]. The 57R marker, linked to the *H1* resistance gene, showed a detection rate of 14.35% in the present study, comparable to the frequencies observed in other potato germplasm surveys [28], further validating the robustness of 57R as a diagnostic marker for *H1*-mediated resistance. The exceptionally low detection rate (3.11%) of *Gro1-4* is largely explained by its wild-species origin (*S. spegazzinii*). Following its transfer into cultivated germplasm, the associated linkage drag may have adversely affected desirable agronomic traits, leading to its progressive elimination from routine breeding populations [30]. The 14.35% detection rate of *H1* observed in this study is substantially lower than the ~80–90% reported in European breeding materials and the ~60% found in Indian cultivars. This disparity likely reflects the differing histories of PCN resistance breeding across regions: European programs initiated systematic screening in the 1970s following widespread PCN outbreaks, whereas China only began such efforts after the first confirmed outbreaks in 2022 [10]. By comparison, the relatively high *Gpa2* frequency (40.43%) in our panel is attributable to the extensive use of CIP germplasm, which has long emphasized *Gpa2*-mediated resistance to *G. pallida*.

When accessions were analyzed according to origin, CIP-introduced materials exhibited significantly greater resistance gene richness than domestically bred lines. Specifically, of the 251 CIP accessions, 146 (58.17%) were positive for *Gpa2*, 38 (15.14%) for *H1*, and 13 (5.18%) for *Gro1-4*. By contrast, among the 118 Chinese breeding lines, only 15 (12.71%) carried *H1*, 20 (16.95%) carried *Gpa2*, and none harbored *Gro1-4*. Of the 10 Yunnan landraces, only two carried *H1* and one carried *Gpa2*. This pattern is consistent with the findings of Asano [31], who reported a markedly higher frequency of the *H1* gene in Japanese-bred cultivars (approximately 46%) than in local landraces, suggesting that systematic resistance breeding programs are effective in accumulating resistance genes. The higher resistance gene content in CIP introductions likely reflects the center’s long-term emphasis on resistance breeding, whereas the scarcity of PCN resistance in Chinese landraces and early self-bred lines may be attributed to the historical absence of PCN resistance as a breeding objective in China prior to the first documented outbreak in 2022.

In the present study, the resistance phenotypes of distinct potato genotypes were comprehensively assessed under field conditions in plots with natural PCN infestation. The *H1*-carrying varieties ‘Dianshu 1208’, ‘Dianshu 14023’, and ‘Dianshu 1520’ were highly resistant, as evidenced by zero cyst counts across all replicates (RF = 0), confirming that the *H1* gene provides highly effective resistance against the Ro1 pathotype of *G. rostochiensis* [32]. Accessions harboring the *Gpa2* gene displayed marked variation in resistance phenotype [33]: the line ‘5S-30’ recorded an RF of 0.37, corresponding to moderate resistance, whereas ‘Dianshu 1209’ recorded an RF of 0.58, exceeding the resistance threshold and thus classified as susceptible. This variation likely reflects the absence of *G. pallida* at the trial site since *Gpa2* confers resistance primarily against the white species [34]. The two gene-pyramided accessions ‘Atlantic’ and ‘Diancaishu 102’ (*H1* + *Gpa2*) likewise reached an RF of 0. The *Gro1-4* marker was detected in only 13 of the 418 accessions, all of which were CIP introductions, and was absent from all Chinese breeding lines and landraces. Although these 13 *Gro1-4*-positive accessions could not be field-evaluated owing to poor local agronomic performance—particularly tuber uniformity and adaptation—their extremely low frequency (3.11%) in the panel remains a notable finding. *Gro1-4* confers broad-spectrum resistance to all known *G. rostochiensis* pathotypes; however, its absence from any Chinese-bred or landrace accession in this study indicates a potential genetic bottleneck in domestic germplasm that may compromise resilience to pathotypes not yet prevalent in China. These results highlight the need for future efforts to introgress *Gro1-4* into locally adapted materials, followed by systematic field validation; its field efficacy after introgression into such backgrounds remains to be confirmed [35]. Nonetheless, the potential of *Gro1-4* as a broad-spectrum resistance source warrants close attention in future research. Currently, the Ro1 pathotype of *G. rostochiensis* is the most widely distributed subspecies among PCN outbreaks in many countries [36]; however, with increasing seed potato movement and international trade, the risk of introducing other pathotypes and the white PCN (*G. pallida*) is growing. Consequently, the scarcity of *Gro1-4* in Chinese germplasm poses a potential risk to the long-term sustainability of PCN resistance breeding in China.

The two gene-pyramided varieties ‘Atlantic’ and ‘Diancaishu 102’ (*H1* + *Gpa2*) recorded an RF of 0, a performance comparable to that of the highly resistant *H1*-only accessions. Under the present experimental conditions, the dual-gene combination (*H1* + *Gpa2*) did not exhibit a detectable advantage over the *H1* single-gene lines, as both groups reached the minimum RF value of 0. Nevertheless, the theoretical benefit of pyramiding both genes lies in its potential to confer broader-spectrum and more durable protection in areas where both *G. rostochiensis* and *G. pallida* are present, as well as in response to the possible future emergence of new virulent pathotypes. Moreover, an experimental evolution study by Lechevalier [37] revealed that adaptation to distinct resistance QTLs involves different genomic regions and is characterized by an absence of cross-virulence, offering a theoretical rationale for deploying gene-pyramiding strategies to achieve broad-spectrum, durable resistance.

Among the accessions screened, 7.42% carried two resistance markers, while none harbored all three, suggesting that gene pyramiding occurs at a relatively low frequency but may nevertheless have important implications for potato breeding. This observation prompts a closer examination of the potential benefits and limitations of pyramiding multiple resistance genes in a single genotype. Theoretical and experimental evidence suggests that stacking multiple resistance loci can broaden resistance spectra and enhance durability, as it imposes stronger and more complex selective constraints on the pathogen population [37]. However, the effectiveness of pyramiding depends on multiple factors, including gene combination, mode of action, and the evolutionary potential of the target pathogen. In this study, the *H1*+*Gpa2* pyramided accession ‘Atlantic’ exhibited complete resistance (RF = 0), comparable to *H1* alone under the tested conditions, indicating that *H1* alone is sufficient for complete protection against the current pathotype. Nevertheless, the theoretical advantage of pyramiding both genes lies in its potential to confer broader-spectrum and more durable resistance in areas where both *G. rostochiensis* and *G. pallida* coexist, and to provide resilience against future emergence of virulent pathotypes. The low frequency of pyramided germplasm in the current Chinese breeding pool (7.42%) underscores the urgent need for systematic efforts to stack diverse resistance alleles into elite cultivars in future breeding programs. Additionally, the presence of multiple resistance genes in breeding materials requires careful management to avoid potential fitness costs or unintended effects on other agronomic traits, although no such negative effects were observed here. Beyond genetic and agronomic considerations, the deployment of resistant cultivars also has implications for food safety and environmental sustainability, as it can reduce reliance on chemical nematicides, thereby lowering chemical residues in potato products and reducing environmental impact. However, the long-term ecological consequences of widespread pyramided cultivar deployment—such as selection pressure on nematode populations—warrant continued monitoring.

While the present study focused on resistance phenotyping based on cyst counts and foliar symptom scoring, we acknowledge that field performance of resistant cultivars also involves additional agronomic traits, including tuber yield, tuber size, and overall plant vigor. Although yield parameters were not systematically quantified in this study, visual assessment indicated that highly resistant genotypes (*H1* and *H1*+*Gpa2*) maintained better above-ground growth and showed no obvious wilting or chlorosis, whereas susceptible genotypes exhibited stunted growth and premature senescence. These observations were consistent with the cyst count data and reproduction factor (RF) values, further supporting the effectiveness of *H1*-mediated resistance. We recognize that the absence of quantitative yield data is a limitation of the present study. Future research should include systematic measurements of tuber yield and quality parameters to fully evaluate the agronomic and economic benefits of PCN-resistant cultivars under field conditions.

The field symptom data (Table 4) showed strong concordance with the RF values determined in this study. The three varieties carrying only the *H1* gene—‘Dianshu 1208’, ‘Dianshu 14023’, and ‘Dianshu 1520’—exhibited no yellowing or wilting symptoms (a score of 0 for both). The two *Gpa2*-only varieties exhibited intermediate symptom levels, with mean scores of 0.5 for both yellowing and wilting. ‘Dianshu 1418’, which lacks any resistance marker, showed a wilting score of 1, and the susceptible control ‘Qingshu 9’ recorded a score of 1 for both symptom types. Notably, neither ‘Atlantic’ nor ‘Diancaishu 102’, both pyramided with *H1* and *Gpa2*, displayed any disease symptoms (a score of 0 for both yellowing and wilting), whereas the susceptible control ‘Qingshu 9’ recorded a score of 1 for both symptoms, further confirming the reliability of the experimental system. These findings validate the utility of field symptom observation as a supplementary tool for PCN resistance evaluation, with visual scoring offering a practical option for preliminary screening in the absence of laboratory-based testing. However, because symptom evaluation is subject to both environmental influence and observer bias, the RF value remains the preferred reference standard for definitive resistance classification [38].

The high detection rate of the *H1* gene in European potato breeding materials [39] is largely attributable to the long-standing emphasis on PCN resistance as a major breeding priority in Europe since the 1970s. Similarly, a study of Indian potato breeding materials [40] found that approximately 60% of tested lines harbored the *H1* gene. By comparison, the detection rates of *H1* (14.35%) and *Gro1-4* (3.11%) observed in this study were markedly lower, which may reflect the delayed commencement and limited historical investment in PCN resistance breeding in China. Nevertheless, the relatively high detection rate of *Gpa2* (40.43%) suggests that CIP-introduced germplasm has contributed substantially to the accumulation of *Gpa2* resistance sources in Chinese breeding pools, providing a promising foundation for systematic resistance breeding efforts in the future.

## 4. Materials and Methods

### 4.1. Experimental Materials

The plant materials comprised 418 potato germplasm accessions collected and maintained by the Institute of Tuber Crops, Yunnan Agricultural University (Appendix A Table A1). 251 from the International Potato Center (CIP, Lima, Peru), 10 from landraces maintained by local farmers in Yunnan Province, 118 from in-house breeding lines preserved at the Institute of Tuber Crops, Yunnan Agricultural University (Kunming, China), and 39 from other Chinese cultivars supplied by various breeding institutions across China. All materials were derived from ex situ germplasm collections; no wild collection was involved, and therefore no specific permits were required. Formal identification was performed by the curators of the respective germplasm collections, and voucher specimens are deposited at the Institute of Tuber Crops, Yunnan Agricultural University (Kunming, China). All experiments complied with institutional and national guidelines. Genomic DNA was extracted from tissue-cultured plantlets of all accessions using the cetyltrimethylammonium bromide (CTAB) method [41]. Based on provenance, the 418 accessions were divided into four groups: CIP introductions (*n* = 251), Yunnan landraces (*n* = 10), breeding lines (*n* = 118), and other Chinese cultivars (*n* = 39). Chi-square and Fisher’s exact tests were used to compare the frequencies of each resistance gene marker among the four groups. For comparative analyses, the 418 accessions were classified into four groups based on their provenance: CIP introductions, Yunnan landraces, breeding lines, and other Chinese cultivars. The frequencies of resistance markers (*H1*/57R, *Gpa2*/Gpa2-2, and *Gro1-4*/Gro1-4) among these groups were analyzed using chi-square tests (for *H1* and *Gpa2*) and Fisher‘s exact test (for *Gro1-4*, due to low expected frequencies). All statistical analyses were performed using IBM SPSS Statistics 25.0, with significance set at *p* < 0.05.

### 4.2. Molecular Marker Screening

Total genomic DNA was extracted from tender leaves of 30-day-old tissue-cultured plantlets using the CTAB method. Three previously published molecular markers, which had been validated in our preliminary experiments, were selected for resistance gene screening: the marker 57R, associated with the *H1* resistance gene(F: 5′-TGCCTGCCTCTCCGATTTCT-3′; R: 5′-GGTTCAGCAAAAGCAAGGACGTG-3′; expected amplicon size of 450 bp) [42]; the marker *Gro1-4*, associated with the *Gro1-4* resistance gene (F: 5′-TCTTTGGAGATACTGATTCTCA-3′; R: 5′-CGACCTAAAATGAAAAGCATCT-3′; 602 bp); and the marker Gpa2-2, associated with the *Gpa2* resistance gene (F: 5′-GCACTTAGAGACTCATTCCA-3′; R: 5′-ACAGATTGTTGGCAGCGAAA-3′; 452 bp) [31].

The PCR reaction mixture (20 µL) consisted of 10 µL of 2× PCR Master Mix, 0.5 µL each of forward and reverse primers (10 µmol/L), 2 µL of template DNA, and ddH2O to a final volume of 20 µL.

The PCR amplification program comprised an initial denaturation at 94 °C for 5 min, followed by 35 cycles of denaturation at 94 °C for 30 s, annealing at 55 °C for 30 s (for 57R/*H1*) or 50 °C for 30 s (for Gpa2-2/*Gpa2* and Gro1-4/*Gro1-4*), and extension at 72 °C for 1 min, with a final extension at 72 °C for 5 min and a final hold at 4 °C until further analysis.

### 4.3. Field Resistance Testing

The field trial was conducted in April 2026 in a field naturally infested with PCN in Xikui Village, Daguan County, Zhaotong City, Yunnan Province, China, at an altitude of 2264 m. The PCN species present at the site was identified as *G. rostochiensis* (golden potato cyst nematode) based on morphological examination and species-specific molecular markers, as previously described [10]. No *G. pallida* (white potato cyst nematode) was detected at the trial location. The field site was confirmed to be naturally infested with *G. rostochiensis* at a level sufficient to cause disease symptoms and cyst formation in susceptible control plants. Thus, the field evaluation in this study specifically targeted resistance against *G. rostochiensis*.

A total of nine potato genotypes were selected for field validation based on the molecular marker screening results, representing four resistance gene categories: (1) *H1* only (3 accessions): ‘Dianshu 1208’, ‘Dianshu 14023’, and ‘Dianshu 1520’; (2) *Gpa2* only (2 accessions): ‘Dianshu 1209’ and ‘5S-30’; (3) both *H1* and *Gpa2* (2 accessions): ‘Atlantic’ and ‘Diancaishu 102’; and (4) marker-free accessions (2 accessions): ‘Dianshu 1418’ (test entry) and ‘Qingshu 9’ (susceptible control).

The experiment was arranged in a randomized complete block design with three replicates per genotype. Plant spacing, row spacing, and water and fertilizer management followed standard local field production practices.

Field surveys were conducted at the early flowering stage. Foliar symptoms (yellowing and wilting) were scored on a 0–3 scale according to the following criteria:

0 = no symptoms;

1 = mild symptoms affecting <25% of leaves;

2 = moderate symptoms affecting 25–50% of leaves;

3 = severe symptoms affecting >50% of leaves or plant death.

The Disease Severity Index (DSI) was calculated as follows:

DSI = (Sum of symptom scores/Total number of plants scored) × 100.

From each plot, three plants were randomly sampled, and the root systems were excavated. Cysts on the roots were counted, and the reproduction factor (RF) was calculated as: RF = Pf/Pi, where Pf is the mean cyst count per root system of the tested accession and Pi is the mean cyst count per root system of the susceptible control ‘Qingshu 9’, based on three plants per accession. Based on internationally accepted criteria [43], resistance was classified as highly resistant (HR, RF < 0.1), moderately resistant (MR, 0.1 ≤ RF < 0.5), or susceptible (S, RF ≥ 0.5). These criteria were applied for resistance classification against *G. rostochiensis* in this study.

### 4.4. Data Processing

Data were organized using Microsoft Excel 2016. Statistical analyses were performed using IBM SPSS Statistics 25.0. Differences in cyst counts among varieties were analyzed using one-way ANOVA. Since Levene’s test indicated unequal variances among groups (*p* < 0.05), Tamhane’s T2 post hoc test was subsequently applied for multiple comparisons. Data are presented as mean ± standard deviation (SD).

## 5. Conclusions

This study yields three main conclusions. First, the germplasm panel screened in this study remains genetically constrained in PCN resistance resources, with strong reliance on *Gpa2* (40.43%) but marked deficiencies in *H1* (14.35%) and *Gro1-4* (3.11%). Second, field validation demonstrated that *H1* provides highly effective and stable resistance against the prevalent *G. rostochiensis* pathotype in southwestern China under the tested conditions, whereas *Gpa2* alone conferred insufficient protection. Third, the five HR lines (RF = 0) identified in this study represent valuable parental materials for resistance breeding.

These findings collectively suggest that future breeding programs should prioritize the introgression of *H1* into locally adapted varieties, supplemented by the introduction of *Gro1-4* using the CIP-derived resistant accessions identified here as donor sources. For farmers, deploying *H1*-carrying cultivars in fields with known *G. rostochiensis* infestation can help minimize yield losses and reduce dependence on chemical nematicides, while regular field monitoring is recommended for early detection. For consumers, the wider adoption of resistant varieties contributes to lower chemical residues in potato products and ensures a more stable supply of high-quality table potatoes, thereby enhancing food safety and market reliability. At the policy level, supporting the development and dissemination of PCN-resistant varieties through breeding programs and extension services, establishing long-term PCN monitoring networks, and allocating resources for research on integrating host resistance with biological control strategies are essential actions. These measures will not only reduce immediate crop losses but also promote long-term soil health and biodiversity by decreasing the environmental footprint of nematicide applications, ultimately underpinning a more sustainable PCN management framework.

## Figures and Tables

**Table 1:** Detection of molecular markers linked to PCN resistance genes in 418 potato accessions.

Resistance Gene	Linked Marker	Positive Accessions (*n*)	Detection Rate (%)
*H1*	57R	60	14.35
*Gpa2*	Gpa2-2	169	40.43
*Gro1-4*	Gro1-4	13	3.11

**Table 2:** Distribution of marker combinations in 418 potato germplasm resources based on molecular marker detection.

Marker Combination	Number of Accessions	Percentage (%)
At least one marker	211	50.48
Two markers	31	7.42
Three markers	0	0.00
None (marker-free)	207	49.52

**Table 3:** Distribution of resistance genes in potato germplasm resources from different sources.

Source Category	Number of Accessions	*H1* (57R)	*Gpa2* (Gpa2-2)	*Gro1-4* (Gro1-4)
CIP introductions	251	38	146	13
Yunnan landraces	10	2	1	0
Breeding lines	118	15	20	0
Other Chinese cultivars	39	5	2	0

Note: For a detailed discussion of the co-occurrence of multiple resistance genes, see Section 3.

**Table 4:** Reproduction factor (RF) and resistance rating of different potato varieties/lines.

Variety/Line	Mean	SD	RF	RF Mean	Resistance Rating
Dianshu 1208	0.00 a	0.00	0.00	0.00	HR
Dianshu 14023	0.00 a	0.00	0.00	0.00	HR
Dianshu 1520	0.00 a	0.00	0.00	0.00	HR
Diancaishu 102	0.00 a	0.00	0.00	0.00	HR
Atlantic	0.00 a	0.00	0.00	0.00	HR
Dianshu 1209	24.67 b	4.04	0.58	0.59	S
5S-30	15.67 b	2.08	0.37	0.38	MR
Dianshu 1418	33.33 c	8.39	0.79	0.80	S
Qingshu 9	42.33 d	5.86	1.00	1.00	S

Note: HR, highly resistant (RF < 0.1); MR, moderately resistant (0.1 ≤ RF < 0.5); S, susceptible (RF ≥ 0.5). Data are presented as mean ± standard deviation (SD) of three biological replicates. Different lowercase letters within the Mean column indicate significant differences among varieties at *p* < 0.05 (one-way ANOVA with Tamhane‘s T2 post hoc test; Levene’s test indicated unequal variances, *p* < 0.05).

**Table 5:** Comparison of damage symptoms among potato varieties/lines with different resistance genotypes.

Variety/Line	Resistance Genotype	Yellowing Severity (0–3 Scale)	Wilting Severity (0–3 Scale)
Dianshu 1208	*H1* only	0.0 ± 0.0 a	0.0 ±0.0 a
Dianshu 14023	*H1* only	0.0 ± 0.0 a	0.0 ±0.0 a
Dianshu 1520	*H1* only	0.0 ± 0.0 a	0.0 ±0.0 a
Dianshu 1209	*Gpa2* only	1.0 ± 1.0 b	0.0 ±0.0 a
5S-30	*Gpa2* only	0.0 ± 0.0 a	1.0 ± 0.0 b
Dianshu 1418	Marker-free	0.0 ± 0.0 a	1.0 ± 0.0 b
Atlantic	*H1* + *Gpa2*	0.0 ± 0.0 a	0.0 ±0.0 a
Diancaishu 102	*H1* + *Gpa2*	0.0 ± 0.0 a	0.0 ±0.0 a
Qingshu 9	Susceptible control	1.0 ± 0.0 b	1.0 ± 1.0 b

Note: Values are mean ± SD of three biological replicates. Scoring scale: 0 = no symptoms, 1 = mild, 2 = moderate. Different lowercase letters within the same column indicate significant differences at *p* < 0.05 (Kruskal–Wallis test followed by post hoc pairwise comparisons).

## Data Availability

The datasets generated and/or analysed during the current study are available in the Figshare repository at https://doi.org/10.6084/m9.figshare.33173870. The dataset contains the molecular marker genotyping results for all 418 potato accessions (Table S1).

## Supplementary Materials

The following supporting information can be downloaded at https://www.mdpi.com/article/10.3390/plants15172686/s1, Table S1: Screening of 418 potato germplasm resources for resistance to PCN; Figure S1: Original, uncropped gel images for Figure 1.

## Abbreviations

The following abbreviations are used in this manuscript:

CIP	International Potato Center
CTAB	Cetyltrimethylammonium bromide
HR	Highly resistant
MR	Moderately resistant
PCN	Potato cyst nematode
RF	Reproduction factor
S	Susceptible

## Appendix A

**Table A1:** List of the 418 potato germplasm accessions used in this study.

No	Accession	Origin	No	Accession	Origin	No	Accession	Origin
1	300055.32	CIP	141	396046.105	CIP	281	19-21-33	Breeding line
2	300063.9	CIP	142	396063.1	CIP	282	BF1869-11	Breeding line
3	300065.4	CIP	143	396063.16	CIP	283	BF1814-13	Breeding line
4	300135.14	CIP	144	396180.22	CIP	284	Longshu 7	Other Chinese cultivars
5	300135.3	CIP	145	396240.2	CIP	285	LU10-36	Other Chinese cultivars
6	300137.31	CIP	146	396240.23	CIP	286	18-22-54	Breeding line
7	301623.15	CIP	147	396241.4	CIP	287	Zinuoxiang	Other Chinese cultivars
8	301624.95	CIP	148	396244.12	CIP	288	19-7-86	Breeding line
9	301026.23	CIP	149	396268.1	CIP	289	BF181-20	Breeding line
10	301037.85	CIP	150	396268.9	CIP	290	BF1810-9	Breeding line
11	301041.26	CIP	151	396269.14	CIP	291	Wotu 5	Other Chinese cultivars
12	301044.36	CIP	152	396269.16	CIP	292	Lucinda	Other Chinese cultivars
13	301045.74	CIP	153	396272.12	CIP	293	Longshu 6	Other Chinese cultivars
14	301055.53	CIP	154	396272.18	CIP	294	Gannongshu 1	Other Chinese cultivars
15	301056.54	CIP	155	396272.2	CIP	295	Xisen 6	Other Chinese cultivars
16	302498.70	CIP	156	396272.21	CIP	296	Diantongshu 1	Breeding line
17	303381.106	CIP	157	396272.37	CIP	297	V7	Other Chinese cultivars
18	303381.30	CIP	158	396273.48	CIP	298	Baishi 2137	Other Chinese cultivars
19	304347.6	CIP	159	396285.1	CIP	299	Maiken	Other Chinese cultivars
20	304349.8	CIP	160	397006.18	CIP	300	Zhongdianhong	Other Chinese cultivars
21	304350.18	CIP	161	397012.20	CIP	301	Atlantic	Other Chinese cultivars
22	304350.78	CIP	162	397039.53	CIP	302	Mozort	Other Chinese cultivars
23	304351.109	CIP	163	397054.3	CIP	303	S15-603	Breeding line
24	304351.31	CIP	164	397060.19	CIP	304	Yunshu 114	Yunnan landrace
25	304,366.46	CIP	165	398098.205	CIP	305	Hongfenjiaren	Other Chinese cultivars
26	304368.22	CIP	166	398098.231	CIP	306	Yunshu 116	Yunnan landrace
27	304371.20	CIP	167	398098.570	CIP	307	BF1772-1	Breeding line
28	304371.58	CIP	168	398098.65	CIP	308	Xisen 8	Other Chinese cultivars
29	304387.17	CIP	169	398180.144	CIP	309	EV	Other Chinese cultivars
30	304387.92	CIP	170	398190.112	CIP	310	Ninglang 3	Yunnan landrace
31	304394.56	CIP	171	398190.200	CIP	311	Ninglang 5	Yunnan landrace
32	304399.15	CIP	172	398190.404	CIP	312	BF1761-4	Breeding line
33	304399.5	CIP	173	398190.523	CIP	313	Lishu 15	Other Chinese cultivars
34	304405.42	CIP	174	398190.530	CIP	314	Huasong 88	Other Chinese cultivars
35	304406.31	CIP	175	398190.571	CIP	315	Zhongdianshu 1	Breeding line
36	374080.5	CIP	176	398190.605	CIP	316	Tianshu 14	Other Chinese cultivars
37	377744.1	CIP	177	398190.615	CIP	317	Longshu 15	Other Chinese cultivars
38	380011.12	CIP	178	398190.735	CIP	318	19-8-7	Breeding line
39	380496.6	CIP	179	398192.41	CIP	319	19-8-3	Breeding line
40	381178.14	CIP	180	398192.553	CIP	320	2022-30-2	Breeding line
41	381379.12	CIP	181	398,92.592	CIP	321	20-3-37	Breeding line
42	381381.9	CIP	182	398193.158	CIP	322	2022-30	Breeding line
43	381403.16	CIP	183	398193.650	CIP	323	BF181-12	Breeding line
44	384321.3	CIP	184	398201.510	CIP	324	Zhongshu 15	Other Chinese cultivars
45	384866.5	CIP	185	398208.29	CIP	325	19-7-14	Breeding line
46	385558.2	CIP	186	398208.505	CIP	326	BF1811-7	Breeding line
47	387164.4	CIP	187	398208.620	CIP	327	Tianshu 11	Other Chinese cultivars
48	389746.2	CIP	188	398208.670	CIP	328	BF1810-7	Breeding line
49	391002.6	CIP	189	399001.44	CIP	329	2022-26-1	Breeding line
50	391011.17	CIP	190	399004.19	CIP	330	BF1815-3	Breeding line
51	391046.14	CIP	191	399048.24	CIP	331	Q9-6	Breeding line
52	391065.81	CIP	192	399049.14	CIP	332	BF1812-3	Breeding line
53	391580.30	CIP	193	399049.16	CIP	333	BF1724-6	Breeding line
54	391585.179	CIP	194	399049.22	CIP	334	17006-2	Breeding line
55	291585.5	CIP	195	399053.11	CIP	335	BF1886-20	Breeding line
56	391691.96	CIP	196	399053.15	CIP	336	BF1814-105	Breeding line
57	392025.7	CIP	197	399062.118	CIP	337	19-17-29	Breeding line
58	392285.72	CIP	198	399067.14	CIP	338	19-8-20	Breeding line
59	392633.64	CIP	199	399067.22	CIP	339	19-8-46	Breeding line
60	392634.49	CIP	200	399072.11	CIP	340	2015-11N69	Breeding line
61	392637.10	CIP	201	399072.28	CIP	341	Dianshu 14009	Breeding line
62	392639.34	CIP	202	399073.23	CIP	342	Dianshu 1504	Breeding line
63	392657.8	CIP	203	399075.32	CIP	343	Dianshu 11-31	Breeding line
64	392821.1	CIP	204	399075.7	CIP	344	18-15-29	Breeding line
65	393075.54	CIP	205	399078.11	CIP	345	Diancaishu 101	Breeding line
66	393077.159	CIP	206	399079.22	CIP	346	20-27-3	Breeding line
67	393077.54	CIP	207	399083.4	CIP	347	19-8-5	Breeding line
68	393079.24	CIP	208	399085.17	CIP	348	BF1845-2	Breeding line
69	393079.4	CIP	209	399085.23	CIP	349	BF1931-4	Breeding line
70	393083.2	CIP	210	399085.30	CIP	350	BF181-6	Breeding line
71	393084.31	CIP	211	694474.16	CIP	351	BF1819-105	Breeding line
72	393220.54	CIP	212	694474.33	CIP	352	19-8-14	Breeding line
73	393242.50	CIP	213	720072	CIP	353	Q9-19-3	Breeding line
74	393248.55	CIP	214	800048	CIP	354	18-6-6	Breeding line
75	393280.57	CIP	215	Yunshu 104	Yunnan landrace	355	BF1836-19	Breeding line
76	393280.82	CIP	216	800959	CIP	356	BF181-2	Breeding line
77	393284.39	CIP	217	I-1085	CIP	357	19-9-12(Red)	Breeding line
78	393339.242	CIP	218	Jizhangshu 12	Other Chinese cultivars	358	18-10(53)	Breeding line
79	393349.68	CIP	219	14-18A	Breeding line	359	14009-23	Breeding line
80	393371.157	CIP	220	12118	Breeding line	360	19-7-29	Breeding line
81	800923	CIP	221	13009-6-11	Breeding line	361	Dianshu 1209	Breeding line
82	393371.58	CIP	222	I-1039	CIP	362	19-9-3	Breeding line
83	393382.44	CIP	223	Dianshu 1418	Breeding line	363	19-8-38	Breeding line
84	393385.39	CIP	224	Dianshu 1208	Breeding line	364	19-9-77	Breeding line
85	393385.47	CIP	225	Hongmeigui	Other Chinese cultivars	365	2-12	Breeding line
86	393399.7	CIP	226	14013-28	Breeding line	366	18-10(129)	Breeding line
87	393536.13	CIP	227	Hezuo 88	Other Chinese cultivars	367	BF1931-40	Breeding line
88	393617.1	CIP	228	108-113	Breeding line	368	19-16-9	Breeding line
89	394223.19	CIP	229	Heimeiren	Other Chinese cultivars	369	19-9-10	Breeding line
90	394223.9	CIP	230	Qingshu 9	Other Chinese cultivars	370	Qingshu 15	Other Chinese cultivars
91	394638.3	CIP	231	14013-127	Breeding line	371	19-16-16	Breeding line
92	394895.7	CIP	232	Gesanghong	Other Chinese cultivars	372	5S-30	Breeding line
93	394898.13	CIP	233	8-3	Breeding line	373	17004-1	Breeding line
94	394899.5	CIP	234	4-2	Breeding line	374	Diancaishu 104	Breeding line
95	394900.1	CIP	235	1153	Breeding line	375	19-8-6	Breeding line
96	395011.2	CIP	236	14013-66	Breeding line	376	19-9-12 (Yellow)	Breeding line
97	395015.6	CIP	237	30-1	Breeding line	377	Qingcaishu 1	Other Chinese cultivars
98	395017.14	CIP	238	14013-35	Breeding line	378	19-9-75	Breeding line
99	395017.227	CIP	239	1131	Breeding line	379	Diancaishu 108	Breeding line
100	395017.229	CIP	240	34-2	Breeding line	380	2015-11-69	Breeding line
101	395017.242	CIP	241	1214	Breeding line	381	BF181-30	Breeding line
102	395037.107	CIP	242	Dianshu 701	Breeding line	382	19-3-20	Breeding line
103	395077.12	CIP	243	1002-1	Breeding line	383	5S-30N79	Breeding line
104	395084.9	CIP	244	5S-16A	Breeding line	384	BF1836-2	Breeding line
105	395096.2	CIP	245	D3	Breeding line	385	703510	CIP
106	395109.29	CIP	246	14008-28	Breeding line	386	705079	CIP
107	395,109.34	CIP	247	54-2	Breeding line	387	703594	CIP
108	395111.13	CIP	248	1-9	Breeding line	388	701025	CIP
109	395112.19	CIP	249	Dianshu1428	Breeding line	389	705,67	CIP
110	395112.32	CIP	250	Dianshu 47	Breeding line	390	703812	CIP
111	395112.36	CIP	251	101-2	Breeding line	391	703546	CIP
112	395112.6	CIP	252	Dianshu 1520	Breeding line	392	704218	CIP
113	395123.6	CIP	253	Laoshuyangyu	Breeding line	393	706768	CIP
114	395169.17	CIP	254	14-6T8	Breeding line	394	703514	CIP
115	395445.16	CIP	255	Lishu 6	Other Chinese cultivars	395	706784	CIP
116	395446.1	CIP	256	5S-133-01	Breeding line	396	703601	CIP
117	395448.1	CIP	257	14-12T19	Breeding line	397	596131.4	CIP
118	396004.225	CIP	258	5S-4A	Breeding line	398	704227	CIP
119	396004.263	CIP	259	Diancaishu 102	Breeding line	399	703567	CIP
120	396004.337	CIP	260	Dianshu 23	Breeding line	400	703508	CIP
121	396008.104	CIP	261	Diancaishu 103	Breeding line	401	703580	CIP
122	396009.240	CIP	262	Guizhouwuyangyu	Other Chinese cultivars	402	703,572	CIP
123	396009.258	CIP	263	1224-2	Breeding line	403	703548	CIP
124	396012.266	CIP	264	Yunshu 304	Yunnan landrace	404	703509	CIP
125	396018.241	CIP	265	Dingshu 4	Other Chinese cultivars	405	703539	CIP
126	396023.109	CIP	266	2022-29-1	Breeding line	406	703570	CIP
127	396026.101	CIP	267	2022-18-2	Breeding line	407	703598	CIP
128	396026.103	CIP	268	14-12T100	Breeding line	408	703581	CIP
129	396027.205	CIP	269	Xuanxiu	Other Chinese cultivars	409	705804	CIP
130	396031.108	CIP	270	Dianshu 14023	Breeding line	410	703559	CIP
131	396031.119	CIP	271	Tianshu 12	Other Chinese cultivars	411	703541	CIP
132	396033.102	CIP	272	Jianchuanhong	Yunnan landrace	412	703897	CIP
133	396034.103	CIP	273	Yunshu 305	Yunnan landrace	413	703545	CIP
134	396034.268	CIP	274	Yunshu 902	Yunnan landrace	414	704120	CIP
135	396036.201	CIP	275	Longshu 10	Other Chinese cultivars	415	703515	CIP
136	396037.215	CIP	276	19-9-15	Breeding line	416	703596	CIP
137	396038.101	CIP	277	2022-29	Breeding line	417	703511	CIP
138	396038.105	CIP	278	Lishu 30	Other Chinese cultivars	418	704228	CIP
139	396038.107	CIP	279	Zhaoshu 12	Other Chinese cultivars			
140	396043.226	CIP	280	19-10-12	Breeding line			

**Table A2:** Screening of 418 potato germplasm for resistance to PCN.

No	Accession	*H1* (57R)	*Gpa2* (Gpa2-2)	*Gro1-4* (Gro1-4)	No	Accession	*H1* (57R)	*Gpa2* (Gpa2-2)	*Gro1-4* (Gro1-4)
1	300055.32	−	−	−	210	399085.30	−	−	−
2	300063.9	−	−	−	211	694474.16	−	+	−
3	300065.4	+	−	−	212	694474.33	+	−	−
4	300135.14	−	+	−	213	720072	−	−	−
5	300135.3	−	−	−	214	800048	−	−	−
6	300137.31	−	+	−	215	Yunshu 104	−	+	−
7	301623.15	−	−	−	216	800959	+	+	−
8	301624.95	+	+	−	217	I−1085	−	−	−
9	301026.23	−	−	−	218	Jizhangshu 12	+	−	−
10	301037.85	−	−	−	219	14–18A	−	−	−
11	301041.26	−	−	−	220	12118	−	+	−
12	301044.36	−	−	−	221	13009-6-11	+	−	−
13	301045.74	−	+	−	222	I-1039	−	−	−
14	301055.53	−	+	−	223	Dianshu 1418	−	−	−
15	301056.54	−	+	−	224	Dianshu 1208	+	−	−
16	302498.70	+	+	−	225	Hongmeigui	−	−	−
17	303381.106	−	+	−	226	14013-28	+	−	−
18	303381.30	−	+	−	227	Hezuo 88	−	−	−
19	304347.6	−	+	−	228	108–113	+	−	−
20	304349.8	−	+	−	229	Heimeiren	−	−	−
21	304350.18	−	+	−	230	Qingshu 9	−	−	−
22	304350.78	−	+	−	231	14013-127	−	−	−
23	304351.109	−	−	−	232	Gesanghong	−	−	−
24	304351.31	−	−	−	233	8-3	−	−	−
25	304366.46	−	+	−	234	4-2	−	−	−
26	304368.22	+	−	−	235	1153	−	−	−
27	304371.20	−	−	−	236	14013-66	−	−	−
28	304371.58	−	+	−	237	30-1	−	−	−
29	304387.17	−	−	−	238	14013-35	−	−	−
30	304387.92	−	+	−	239	1131	−	−	−
31	304394.56	+	+	−	240	34-2	−	+	−
32	304399.15	−	+	−	241	1214	−	+	−
33	304399.5	−	+	−	242	Dianshu 701	−	+	−
34	304405.42	−	+	−	243	1002-1	−	+	−
35	304406.31	−	+	−	244	5S-16A	−	−	−
36	374080.5	−	+	−	245	D3	−	−	−
37	377744.1	−	−	−	246	14008-28	−	+	−
38	380011.12	−	−	−	247	54-2	+	−	−
39	380496.6	−	−	−	248	1-9	+	−	−
40	381178.14	−	−	−	249	Dianshu 1428	−	+	−
41	381379.12	−	+	−	250	Dianshu 47	−	−	−
42	381381.9	−	+	−	251	101-2	−	−	−
43	381403.16	−	+	−	252	Dianshu 1520	+	−	−
44	384321.3	−	−	−	253	Laoshuyangyu	−	−	−
45	384866.5	−	−	−	254	14-6T8	−	−	−
46	385558.2	−	−	−	255	Li Shu 6	−	−	−
47	387164.4	−	+	−	256	5S-133-01	−	−	−
48	389746.2	−	+	−	257	14-12T19	−	+	−
49	391002.6	−	−	−	258	5S-4A	+	−	−
50	391011.17	−	−	−	259	Diancaishu 102	+	+	−
51	391046.14	−	−	−	260	Dianshu 23	−	−	−
52	391065.81	−	−	−	261	Diancaishu 103	−	−	−
53	391580.30	−	−	−	262	Guizhouwuyangyu	−	−	−
54	391585.179	−	−	−	263	1224-2	+	−	−
55	291585.5	+	−	−	264	Yunshu 304	+	−	−
56	391691.96	−	−	−	265	Dingshu 4	−	−	−
57	392025.7	−	+	−	266	2022-29-1	−	−	−
58	392285.72	−	+	−	267	2022-18-2	−	−	−
59	392633.64	−	+	−	268	14-12T100	−	+	−
60	392634.49	−	−	−	269	Xuanxiu	−	−	−
61	392637.10	−	−	−	270	Dianshu 14023	+	−	−
62	392639.34	−	+	−	271	Tianshu 12	−	−	−
63	392657.8	+	+	−	272	Jianchuanhong	−	−	−
64	392821.1	−	−	−	273	Yunshu 305	−	−	−
65	393075.54	−	+	−	274	Yunshu 902	+	−	−
66	393077.159	−	+	−	275	Longshu 10	−	−	−
67	393077.54	−	+	−	276	19-9-15	−	−	−
68	393079.24	−	+	−	277	2022-29	−	−	−
69	393079.4	−	+	−	278	Lishu 30	−	−	−
70	393083.2	−	−	−	279	Zhaoshu 12	−	−	−
71	393084.31	−	+	−	280	19-10-12	−	−	−
72	393220.54	−	−	−	281	19-21-33	−	−	−
73	393242.50	−	−	−	282	BF1869-11	−	−	−
74	393248.55	+	−	−	283	BF1814-13	−	−	−
75	393280.57	+	−	−	284	Longshu 7	−	−	−
76	393280.82	−	−	−	285	LU10-36	−	−	−
77	393284.39	+	−	−	286	18-22-54	−	−	−
78	393339.242	−	−	−	287	Zinuoxiang	−	−	−
79	393349.68	−	−	−	288	19-7-86	−	−	−
80	393371.157	−	+	−	289	BF181-20	−	−	−
81	800923	−	−	−	290	BF1810-9	−	−	−
82	393371.58	−	+	−	291	Wotu 5	−	−	−
83	393382.44	−	−	−	292	Lucinda	−	−	−
84	393385.39	−	−	−	293	Longshu 6	+	−	−
85	393385.47	−	−	−	294	Gannongshu 1	−	−	−
86	393399.7	−	−	−	295	Xisen 6	+	−	−
87	393536.13	−	+	−	296	Diantongshu 1	−	−	−
88	393617.1	−	+	−	297	V7	−	−	−
89	394223.19	−	+	−	298	Baishi 2137	−	−	−
90	394223.9	−	+	−	299	Maiken	−	−	−
91	394638.3	+	−	−	300	Zhongdianhong	−	−	−
92	394895.7	−	+	−	301	Atlantic	+	+	−
93	394898.13	−	−	−	302	Mozort	−	−	−
94	394899.5	−	−	−	303	S15-603	−	−	−
95	394900.1	−	+	−	304	Yunshu 114	−	−	−
96	395011.2	−	+	−	305	Hongfenjiaren	−	−	−
97	395015.6	−	−	−	306	Yunshu 116	−	−	−
98	395017.14	−	+	−	307	BF1772-1	−	−	−
99	395017.227	+	−	−	308	Xisen 8	−	−	−
100	395017.229	−	+	−	309	EV	+	−	−
101	395017.242	−	+	−	310	Ninglang 3	−	−	−
102	395037.107	−	+	−	311	Ninglang 5	−	−	−
103	395077.12	−	−	−	312	BF1761-4	−	−	−
104	395084.9	−	−	−	313	Lishu 15	−	−	−
105	395096.2	−	+	−	314	Huasong 88	−	−	−
106	395109.29	−	+	−	315	Zhongdianshu 1	−	−	−
107	395109.34	−	+	−	316	Tianshu 14	−	−	−
108	395111.13	−	+	−	317	Longshu 15	−	+	−
109	395112.19	−	+	−	318	19-8-7	−	−	−
110	395112.32	−	+	−	319	19-8-3	−	−	−
111	395112.36	−	+	−	320	2022-30-2	−	−	−
112	395112.6	−	+	−	321	20-3-37	−	−	−
113	395123.6	−	+	−	322	2022-30	−	+	−
114	395169.17	−	−	−	323	BF181-12	−	−	−
115	395445.16	+	+	−	324	Zhongshu 15	−	−	−
116	395446.1	−	+	−	325	19-7-14	−	−	−
117	395448.1	−	+	−	326	BF1811-7	−	−	−
118	396004.225	−	−	−	327	Tianshu 11	−	−	−
119	396004.263	−	−	−	328	BF1810-7	−	−	−
120	396004.337	−	−	−	329	2022-26-1	−	−	−
121	396008.104	−	−	−	330	BF1815-3	−	−	−
122	396009.240	+	−	−	331	Q9-6	−	−	−
123	396009.258	+	−	−	332	BF1812-3	−	−	−
124	396012.266	−	−	−	333	BF1724-6	−	+	−
125	396018.241	+	−	−	334	17006-2	−	−	−
126	396023.109	−	−	−	335	BF1886-20	−	−	−
127	396026.101	−	−	−	336	BF1814-105	−	−	−
128	396026.103	−	+	−	337	19-17-29	−	−	−
129	396027.205	−	+	−	338	19-8-20	−	−	−
130	396031.108	−	−	−	339	19-8-46	−	−	−
131	396031.119	−	−	−	340	2015-11N69	−	−	−
132	396033.102	−	+	−	341	Dianshu 14009	+	−	−
133	396034.103	+	−	−	342	Dianshu 1504	−	−	−
134	396034.268	−	+	−	343	Dianshu 11-31	−	−	−
135	396036.201	−	−	−	344	18-15-29	−	−	−
136	396037.215	−	+	−	345	Diancaishu 101	−	−	−
137	396038.101	−	−	−	346	20-27-3	−	−	−
138	396038.105	−	+	−	347	19-8-5	−	−	−
139	396038.107	−	+	−	348	BF1845-2	−	+	−
140	396043.226	−	−	−	349	BF1931-4	−	−	−
141	396046.105	−	−	−	350	BF181-6	−	−	−
142	396063.1	−	+	−	351	BF1819-105	−	−	−
143	396063.16	−	+	−	352	19-8-14	−	+	−
144	396180.22	−	+	−	353	Q9-19-3	−	−	−
145	396240.2	−	−	+	354	18-6-6	−	−	−
146	396240.23	−	−	−	355	BF1836-19	−	−	−
147	396241.4	−	+	−	356	BF181-2	−	−	−
148	396244.12	−	−	−	357	19-9-12(Red)	−	−	−
149	396268.1	−	+	−	358	18-10(53)	−	−	−
150	396268.9	−	+	−	359	14009-23	+	−	−
151	396269.14	−	+	−	360	19-7-29	−	−	−
152	396269.16	−	+	−	361	Dianshu 1209	−	+	−
153	396272.12	−	+	−	362	19-9-3	−	−	−
154	396272.18	−	+	−	363	19-8-38	−	−	−
155	396272.2	−	+	−	364	19-9-77	+	−	−
156	396272.21	−	−	−	365	2-12	−	−	−
157	396272.37	−	+	−	366	18-10(129)	−	−	−
158	396273.48	−	+	−	367	BF1931-40	−	+	−
159	396285.1	−	+	−	368	19-16-9	−	−	−
160	397006.18	−	+	−	369	19-9-10	−	+	−
161	397012.20	−	+	−	370	Qingshu 15	−	−	−
162	397039.53	−	+	−	371	19-16-16	−	−	−
163	397054.3	+	+	−	372	5S-30	−	+	−
164	397060.19	−	+	−	373	17004-1	−	+	−
165	398098.205	−	+	−	374	Diancaishu 104	−	+	−
166	398,98.231	−	−	−	375	19-8-6	−	−	−
167	398098.570	−	−	−	376	19-9-12 (Yellow)	−	−	−
168	398098.65	−	+	−	377	Qingcaishu 1	−	−	−
169	398180.144	−	+	−	378	19-9-75	−	−	−
170	398190.112	−	−	−	379	Diancaishu 108	−	−	−
171	398190.200	−	+	−	380	2015-11-69	−	−	−
172	398190.404	−	+	−	381	BF181-30	−	−	−
173	398,90.523	−	+	−	382	19-3-20	−	−	−
174	398190.530	−	−	−	383	5S-30N79	−	−	−
175	398190.571	−	+	−	384	BF1836-2	+	−	−
176	398190.605	−	+	−	385	703510	−	+	−
177	398190.615	−	−	−	386	705079	−	+	−
178	398190.735	−	+	−	387	703594	−	+	−
179	398192.41	−	+	−	388	701025	−	+	−
180	398192.553	−	+	−	389	705167	−	+	−
181	398192.592	−	+	−	390	703812	−	+	−
182	398193.158	−	+	−	391	703546	−	−	−
183	398,93.650	−	+	−	392	704218	−	+	−
184	398201.510	−	+	−	393	706768	−	−	−
185	398208.29	−	+	−	394	703514	−	+	−
186	398208.505	−	+	−	395	706784	+	−	−
187	398208.620	−	+	−	396	703601	+	−	−
188	398,08.670	−	−	−	397	596131.4	+	−	−
189	399001.44	−	+	+	398	704227	+	+	−
190	399004.19	−	+	+	399	703567	+	+	−
191	399048.24	−	+	−	400	703508	+	−	−
192	399049.14	−	+	+	401	703580	+	+	−
193	399049.16	−	+	−	402	703572	+	+	−
194	399049.22	−	+	+	403	703548	+	+	−
195	399053.11	−	+	−	404	703509	+	+	−
196	399053.15	−	+	+	405	703539	+	+	−
197	399062.118	−	+	+	406	703570	+	+	−
198	399067.14	−	−	+	407	703598	+	+	−
199	399067.22	−	+	+	408	703581	+	+	−
200	399072.11	−	−	−	409	705804	+	+	−
201	399072.28	−	−	+	410	703559	−	+	−
202	399073.23	−	+	+	411	703541	−	+	−
203	399075.32	−	+	+	412	703897	−	−	−
204	399075.7	−	+	−	413	703545	+	+	−
205	399078.11	−	+	−	414	704120	−	−	−
206	399079.22	−	−	−	415	703515	−	−	−
207	399083.4	−	+	+	416	703596	+	−	−
208	399085.17	−	−	−	417	703511	−	−	−
209	399085.23	−	−	−	418	704228	+	−	−

Note: + indicates the presence of the resistance gene; − indicates the absence of the resistance gene.
