# Supplementary material for: Molecular Screening and Field Validation of Potato Germplasm for Resistance to Potato Cyst Nematodes
Source: Plants (Basel). 2026 Sep 1;15(17):2686. doi: 10.3390/plants15172686 (PMC13567405; doi:10.3390/plants15172686)
Supplement: Supplementary file 1 [file plants-15-02686-s001.zip › plants-4526810-supplementary.pdf]

Table S1

No	Accession	Origin	*H1*(57R)	*Gpa2*(Gpa2-2)	*Gro1-4*(Gro1-4)
1	300055.32	CIP	-	-	-
2	300063.9	CIP	-	-	-
3	300065.4	CIP	+	-	-
4	300135.14	CIP	-	+	-
5	300135.3	CIP	-	-	-
6	300137.31	CIP	-	+	-
7	301623.15	CIP	-	-	-
8	301624.95	CIP	+	+	-
9	301026.23	CIP	-	-	-
10	301037.85	CIP	-	-	-
11	301041.26	CIP	-	-	-
12	301044.36	CIP	-	-	-
13	301045.74	CIP	-	+	-
14	301055.53	CIP	-	+	-
15	301056.54	CIP	-	+	-
16	302498.7	CIP	+	+	-
17	303381.106	CIP	-	+	-
18	303381.3	CIP	-	+	-
19	304347.6	CIP	-	+	-
20	304349.8	CIP	-	+	-
21	304350.18	CIP	-	+	-
22	304350.78	CIP	-	+	-
23	304351.109	CIP	-	-	-
24	304351.31	CIP	-	-	-
25	304366.46	CIP	-	+	-
26	304368.22	CIP	+	-	-
27	304371.2	CIP	-	-	-
28	304371.58	CIP	-	+	-
29	304387.17	CIP	-	-	-
30	304387.92	CIP	-	+	-
31	304394.56	CIP	+	+	-
32	304399.15	CIP	-	+	-
33	304399.5	CIP	-	+	-
34	304405.42	CIP	-	+	-
35	304406.31	CIP	-	+	-
36	374080.5	CIP	-	+	-
37	377744.1	CIP	-	-	-
38	380011.12	CIP	-	-	-
39	380496.6	CIP	-	-	-
40	381178.14	CIP	-	-	-
41	381379.12	CIP	-	+	-
42	381381.9	CIP	-	+	-
43	381403.16	CIP	-	+	-
44	384321.3	CIP	-	-	-
45	384866.5	CIP	-	-	-
46	385558.2	CIP	-	-	-
47	387164.4	CIP	-	+	-
48	389746.2	CIP	-	+	-
49	391002.6	CIP	-	-	-
50	391011.17	CIP	-	-	-
51	391046.14	CIP	-	-	-
52	391065.81	CIP	-	-	-

53	391580.3	CIP	-	-	-
54	391585.179	CIP	-	-	-
55	291585.5	CIP	+	-	-
56	391691.96	CIP	-	-	-
57	392025.7	CIP	-	+	-
58	392285.72	CIP	-	+	-
59	392633.64	CIP	-	+	-
60	392634.49	CIP	-	-	-
61	392637.1	CIP	-	-	-
62	392639.34	CIP	-	+	-
63	392657.8	CIP	+	+	-
64	392821.1	CIP	-	-	-
65	393075.54	CIP	-	+	-
66	393077.159	CIP	-	+	-
67	393077.54	CIP	-	+	-
68	393079.24	CIP	-	+	-
69	393079.4	CIP	-	+	-
70	393083.2	CIP	-	-	-
71	393084.31	CIP	-	+	-
72	393220.54	CIP	-	-	-
73	393242.5	CIP	-	-	-
74	393248.55	CIP	+	-	-
75	393280.57	CIP	+	-	-
76	393280.82	CIP	-	-	-
77	393284.39	CIP	+	-	-
78	393339.242	CIP	-	-	-
79	393349.68	CIP	-	-	-
80	393371.157	CIP	-	+	-
81	800923	CIP	-	-	-
82	393371.58	CIP	-	+	-
83	393382.44	CIP	-	-	-
84	393385.39	CIP	-	-	-
85	393385.47	CIP	-	-	-
86	393399.7	CIP	-	-	-
87	393536.13	CIP	-	+	-
88	393617.1	CIP	-	+	-
89	394223.19	CIP	-	+	-
90	394223.9	CIP	-	+	-
91	394638.3	CIP	+	-	-
92	394895.7	CIP	-	+	-
93	394898.13	CIP	-	-	-
94	394899.5	CIP	-	-	-
95	394900.1	CIP	-	+	-
96	395011.2	CIP	-	+	-
97	395015.6	CIP	-	-	-
98	395017.14	CIP	-	+	-
99	395017.227	CIP	+	-	-
100	395017.229	CIP	-	+	-
101	395017.242	CIP	-	+	-
102	395037.107	CIP	-	+	-
103	395077.12	CIP	-	-	-
104	395084.9	CIP	-	-	-
105	395096.2	CIP	-	+	-
106	395109.29	CIP	-	+	-

107	395109.34	CIP	-	+	-
108	395111.13	CIP	-	+	-
109	395112.19	CIP	-	+	-
110	395112.32	CIP	-	+	-
111	395112.36	CIP	-	+	-
112	395112.6	CIP	-	+	-
113	395123.6	CIP	-	+	-
114	395169.17	CIP	-	-	-
115	395445.16	CIP	+	+	-
116	395446.1	CIP	-	+	-
117	395448.1	CIP	-	+	-
118	396004.225	CIP	-	-	-
119	396004.263	CIP	-	-	-
120	396004.337	CIP	-	-	-
121	396008.104	CIP	-	-	-
122	396009.24	CIP	+	-	-
123	396009.258	CIP	+	-	-
124	396012.266	CIP	-	-	-
125	396018.241	CIP	+	-	-
126	396023.109	CIP	-	-	-
127	396026.101	CIP	-	-	-
128	396026.103	CIP	-	+	-
129	396027.205	CIP	-	+	-
130	396031.108	CIP	-	-	-
131	396031.119	CIP	-	-	-
132	396033.102	CIP	-	+	-
133	396034.103	CIP	+	-	-
134	396034.268	CIP	-	+	-
135	396036.201	CIP	-	-	-
136	396037.215	CIP	-	+	-
137	396038.101	CIP	-	-	-
138	396038.105	CIP	-	+	-
139	396038.107	CIP	-	+	-
140	396043.226	CIP	-	-	-
141	396046.105	CIP	-	-	-
142	396063.1	CIP	-	+	-
143	396063.16	CIP	-	+	-
144	396180.22	CIP	-	+	-
145	396240.2	CIP	-	-	+
146	396240.23	CIP	-	-	-
147	396241.4	CIP	-	+	-
148	396244.12	CIP	-	-	-
149	396268.1	CIP	-	+	-
150	396268.9	CIP	-	+	-
151	396269.14	CIP	-	+	-
152	396269.16	CIP	-	+	-
153	396272.12	CIP	-	+	-
154	396272.18	CIP	-	+	-
155	396272.2	CIP	-	+	-
156	396272.21	CIP	-	-	-
157	396272.37	CIP	-	+	-
158	396273.48	CIP	-	+	-
159	396285.1	CIP	-	+	-
160	397006.18	CIP	-	+	-

161	397012.2	CIP	-	+	-
162	397039.53	CIP	-	+	-
163	397054.3	CIP	+	+	-
164	397060.19	CIP	-	+	-
165	398098.205	CIP	-	+	-
166	398098.231	CIP	-	-	-
167	398098.57	CIP	-	-	-
168	398098.65	CIP	-	+	-
169	398180.144	CIP	-	+	-
170	398190.112	CIP	-	-	-
171	398190.2	CIP	-	+	-
172	398190.404	CIP	-	+	-
173	398190.523	CIP	-	+	-
174	398190.53	CIP	-	-	-
175	398190.571	CIP	-	+	-
176	398190.605	CIP	-	+	-
177	398190.615	CIP	-	-	-
178	398190.735	CIP	-	+	-
179	398192.41	CIP	-	+	-
180	398192.553	CIP	-	+	-
181	398192.592	CIP	-	+	-
182	398193.158	CIP	-	+	-
183	398193.65	CIP	-	+	-
184	398201.51	CIP	-	+	-
185	398208.29	CIP	-	+	-
186	398208.505	CIP	-	+	-
187	398208.62	CIP	-	+	-
188	398208.67	CIP	-	-	-
189	399001.44	CIP	-	+	+
190	399004.19	CIP	-	+	+
191	399048.24	CIP	-	+	-
192	399049.14	CIP	-	+	+
193	399049.16	CIP	-	+	-
194	399049.22	CIP	-	+	+
195	399053.11	CIP	-	+	-
196	399053.15	CIP	-	+	+
197	399062.118	CIP	-	+	+
198	399067.14	CIP	-	-	+
199	399067.22	CIP	-	+	+
200	399072.11	CIP	-	-	-
201	399072.28	CIP	-	-	+
202	399073.23	CIP	-	+	+
203	399075.32	CIP	-	+	+
204	399075.7	CIP	-	+	-
205	399078.11	CIP	-	+	-
206	399079.22	CIP	-	-	-
207	399083.4	CIP	-	+	+
208	399085.17	CIP	-	-	-
209	399085.23	CIP	-	-	-
210	399085.3	CIP	-	-	-
211	694474.16	CIP	-	+	-
212	694474.33	CIP	+	-	-
213	720072	CIP	-	-	-
214	800048	CIP	-	-	-

215	Yunshu 104	Yunnan landrace	-	+	-
216	800959	CIP	+	+	-
217	I-1085	CIP	-	-	-
218	Jizhangshu 12	Other Chinese cultivars	+	-	-
219	14–18A	Breeding line	-	-	-
220	12118	Breeding line	-	+	-
221	13009-6-11	Breeding line	+	-	-
222	I-1039	CIP	-	-	-
223	Dianshu 1418	Breeding line	-	-	-
224	Dianshu 1208	Breeding line	+	-	-
225	Hongmeigui	Other Chinese cultivars	-	-	-
226	14013-28	Breeding line	+	-	-
227	Hezuo 88	Other Chinese cultivars	-	-	-
228	108–113	Breeding line	+	-	-
229	Heimeiren	Other Chinese cultivars	-	-	-
230	Qingshu 9	Other Chinese cultivars	-	-	-
231	14013-127	Breeding line	-	-	-
232	Gesanghong	Other Chinese cultivars	-	-	-
233	8-3	Breeding line	-	-	-
234	4-2	Breeding line	-	-	-
235	1153	Breeding line	-	-	-
236	14013-66	Breeding line	-	-	-
237	30-1	Breeding line	-	-	-
238	14013-35	Breeding line	-	-	-
239	1131	Breeding line	-	-	-
240	34-2	Breeding line	-	+	-
241	1214	Breeding line	-	+	-
242	Dianshu 701	Breeding line	-	+	-
243	1002-1	Breeding line	-	+	-
244	5S-16A	Breeding line	-	-	-
245	D3	Breeding line	-	-	-
246	14008-28	Breeding line	-	+	-
247	54-2	Breeding line	+	-	-
248	1-9	Breeding line	+	-	-
249	Dianshu 1428	Breeding line	-	+	-
250	Dianshu 47	Breeding line	-	-	-
251	101-2	Breeding line	-	-	-
252	Dianshu 1520	Breeding line	+	-	-
253	Laoshuyangyu	Breeding line	-	-	-
254	14-6T8	Breeding line	-	-	-
255	Li Shu 6	Other Chinese cultivars	-	-	-
256	5S-133-01	Breeding line	-	-	-
257	14-12T19	Breeding line	-	+	-
258	5S-4A	Breeding line	+	-	-
259	Diancaishu 102	Breeding line	+	+	-
260	Dianshu 23	Breeding line	-	-	-
261	Diancaishu 103	Breeding line	-	-	-
262	Guizhouwuyangyu	Other Chinese cultivars	-	-	-
263	1224-2	Breeding line	+	-	-
264	Yunshu 304	Yunnan landrace	+	-	-
265	Dingshu 4	Other Chinese cultivars	-	-	-
266	2022-29-1	Breeding line	-	-	-
267	2022-18-2	Breeding line	-	-	-
268	14-12T100	Breeding line	-	+	-

269	Xuanxiu	Other Chinese cultivars	-	-	-
270	Dianshu 14023	Breeding line	+	-	-
271	Tianshu 12	Other Chinese cultivars	-	-	-
272	Jianchuanhong	Yunnan landrace	-	-	-
273	Yunshu 305	Yunnan landrace	-	-	-
274	Yunshu 902	Yunnan landrace	+	-	-
275	Longshu 10	Other Chinese cultivars	-	-	-
276	19-9-15	Breeding line	-	-	-
277	2022-29	Breeding line	-	-	-
278	Lishu 30	Other Chinese cultivars	-	-	-
279	Zhaoshu 12	Other Chinese cultivars	-	-	-
280	19-10-12	Breeding line	-	-	-
281	19-21-33	Breeding line	-	-	-
282	BF1869-11	Breeding line	-	-	-
283	BF1814-13	Breeding line	-	-	-
284	Longshu 7	Other Chinese cultivars	-	-	-
285	LU10-36	Other Chinese cultivars	-	-	-
286	18-22-54	Breeding line	-	-	-
287	Zinuoxiang	Other Chinese cultivars	-	-	-
288	19-7-86	Breeding line	-	-	-
289	BF181-20	Breeding line	-	-	-
290	BF1810-9	Breeding line	-	-	-
291	Wotu 5	Other Chinese cultivars	-	-	-
292	Lucinda	Other Chinese cultivars	-	-	-
293	Longshu 6	Other Chinese cultivars	+	-	-
294	Gannongshu 1	Other Chinese cultivars	-	-	-
295	Xisen 6	Other Chinese cultivars	+	-	-
296	Diantongshu 1	Breeding line	-	-	-
297	V7	Other Chinese cultivars	-	-	-
298	Baishi 2137	Other Chinese cultivars	-	-	-
299	Maiken	Other Chinese cultivars	-	-	-
300	Zhongdianhong	Other Chinese cultivars	-	-	-
301	Atlantic	Other Chinese cultivars	+	+	-
302	Mozort	Other Chinese cultivars	-	-	-
303	S15-603	Breeding line	-	-	-
304	Yunshu 114	Yunnan landrace	-	-	-
305	Hongfenjiaren	Other Chinese cultivars	-	-	-
306	Yunshu 116	Yunnan landrace	-	-	-
307	BF1772-1	Breeding line	-	-	-
308	Xisen 8	Other Chinese cultivars	-	-	-
309	EV	Other Chinese cultivars	+	-	-
310	Ninglang 3	Yunnan landrace	-	-	-
311	Ninglang 5	Yunnan landrace	-	-	-
312	BF1761-4	Breeding line	-	-	-
313	Lishu 15	Other Chinese cultivars	-	-	-
314	Huasong 88	Other Chinese cultivars	-	-	-
315	Zhongdianshu 1	Breeding line	-	-	-
316	Tianshu 14	Other Chinese cultivars	-	-	-
317	Longshu 15	Other Chinese cultivars	-	+	-
318	19-8-7	Breeding line	-	-	-
319	19-8-3	Breeding line	-	-	-
320	2022-30-2	Breeding line	-	-	-
321	20-3-37	Breeding line	-	-	-
322	2022-30	Breeding line	-	+	-

323	BF181-12	Breeding line	-	-	-
324	Zhongshu 15	Other Chinese cultivars	-	-	-
325	19-7-14	Breeding line	-	-	-
326	BF1811-7	Breeding line	-	-	-
327	Tianshu 11	Other Chinese cultivars	-	-	-
328	BF1810-7	Breeding line	-	-	-
329	2022-26-1	Breeding line	-	-	-
330	BF1815-3	Breeding line	-	-	-
331	Q9-6	Breeding line	-	-	-
332	BF1812-3	Breeding line	-	-	-
333	BF1724-6	Breeding line	-	+	-
334	17006-2	Breeding line	-	-	-
335	BF1886-20	Breeding line	-	-	-
336	BF1814-105	Breeding line	-	-	-
337	19-17-29	Breeding line	-	-	-
338	19-8-20	Breeding line	-	-	-
339	19-8-46	Breeding line	-	-	-
340	2015-11N69	Breeding line	-	-	-
341	Dianshu 14009	Breeding line	+	-	-
342	Dianshu 1504	Breeding line	-	-	-
343	Dianshu 11-31	Breeding line	-	-	-
344	18-15-29	Breeding line	-	-	-
345	Diancaishu 101	Breeding line	-	-	-
346	20-27-3	Breeding line	-	-	-
347	19-8-5	Breeding line	-	-	-
348	BF1845-2	Breeding line	-	+	-
349	BF1931-4	Breeding line	-	-	-
350	BF181-6	Breeding line	-	-	-
351	BF1819-105	Breeding line	-	-	-
352	19-8-14	Breeding line	-	+	-
353	Q9-19-3	Breeding line	-	-	-
354	18-6-6	Breeding line	-	-	-
355	BF1836-19	Breeding line	-	-	-
356	BF181-2	Breeding line	-	-	-
357	19-9-12(Red)	Breeding line	-	-	-
358	18-10(53)	Breeding line	-	-	-
359	14009-23	Breeding line	+	-	-
360	19-7-29	Breeding line	-	-	-
361	Dianshu 1209	Breeding line	-	+	-
362	19-9-3	Breeding line	-	-	-
363	19-8-38	Breeding line	-	-	-
364	19-9-77	Breeding line	+	-	-
365	2-12	Breeding line	-	-	-
366	18-10(129)	Breeding line	-	-	-
367	BF1931-40	Breeding line	-	+	-
368	19-16-9	Breeding line	-	-	-
369	19-9-10	Breeding line	-	+	-
370	Qingshu 15	Other Chinese cultivars	-	-	-
371	19-16-16	Breeding line	-	-	-
372	5S-30	Breeding line	-	+	-
373	17004-1	Breeding line	-	+	-
374	Diancaishu 104	Breeding line	-	+	-
375	19-8-6	Breeding line	-	-	-
376	19-9-12 (Yellow)	Breeding line	-	-	-

377	Qingcaishu 1	Other Chinese cultivars	-	-	-
378	19-9-75	Breeding line	-	-	-
379	Diancaishu 108	Breeding line	-	-	-
380	2015-11-69	Breeding line	-	-	-
381	BF181-30	Breeding line	-	-	-
382	19-3-20	Breeding line	-	-	-
383	5S-30N79	Breeding line	-	-	-
384	BF1836-2	Breeding line	+	-	-
385	703510	CIP	-	+	-
386	705079	CIP	-	+	-
387	703594	CIP	-	+	-
388	701025	CIP	-	+	-
389	705167	CIP	-	+	-
390	703812	CIP	-	+	-
391	703546	CIP	-	-	-
392	704218	CIP	-	+	-
393	706768	CIP	-	-	-
394	703514	CIP	-	+	-
395	706784	CIP	+	-	-
396	703601	CIP	+	-	-
397	596131.4	CIP	+	-	-
398	704227	CIP	+	+	-
399	703567	CIP	+	+	-
400	703508	CIP	+	-	-
401	703580	CIP	+	+	-
402	703572	CIP	+	+	-
403	703548	CIP	+	+	-
404	703509	CIP	+	+	-
405	703539	CIP	+	+	-
406	703570	CIP	+	+	-
407	703598	CIP	+	+	-
408	703581	CIP	+	+	-
409	705804	CIP	+	+	-
410	703559	CIP	-	+	-
411	703541	CIP	-	+	-
412	703897	CIP	-	-	-
413	703545	CIP	+	+	-
414	704120	CIP	-	-	-
415	703515	CIP	-	-	-
416	703596	CIP	+	-	-
417	703511	CIP	-	-	-
418	704228	CIP	+	-	-

Note: + indicates the presence of resistance gene; – indicates the absence of resistance gene.

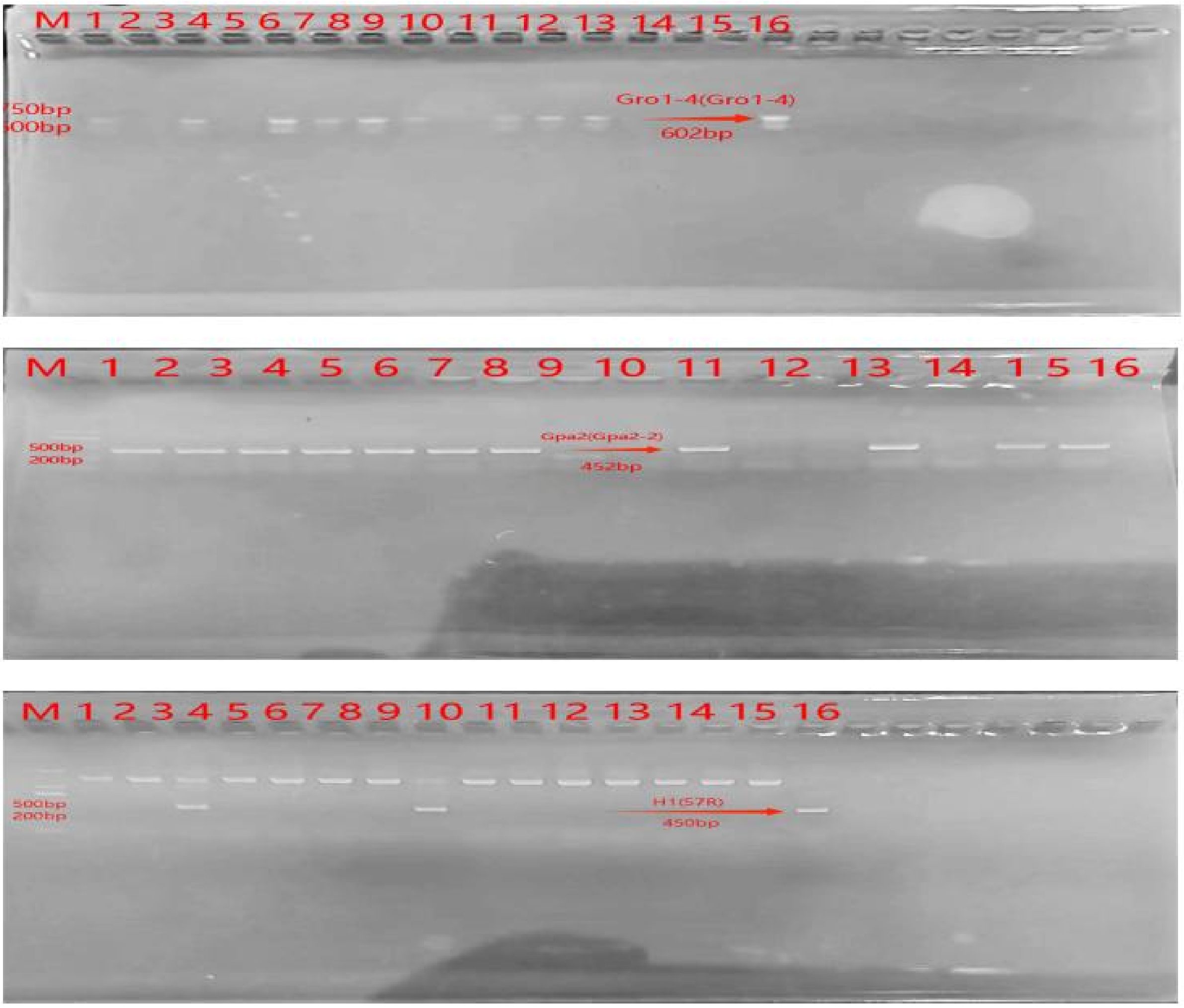

Figure S1. PCR detection of three resistance gene markers in potato germplasm. **M:** BM 2000+ DNA Marker. Lanes 1–16: *Gro1-4* gene detection (Gro1-4 marker). 1: 399049.14; 2: 399049.16; 3: 399049.22; 4: 399053.11; 5: 399053.15; 6: 399062.118; 7: 399067.14; 8: 399067.22; 9: 399072.11; 10: 399072.28; 11: 399073.23; 12: 399075.32; 13: 399075.7; 14: 399078.11; 15: 399079.22; 16: 399083.4. Lanes 1–16: *Gpa2* gene detection (Gpa2-2 marker). 1: 302498.70; 2: 303381.106; 3: 303381.30; 4: 304347.6; 5: 304349.8; 6: 304350.18; 7: 304350.78; 8: 304351.109; 9: 304351.31; 10: 304366.46; 11: 304368.22; 12: 304371.20; 13: 304371.58; 14: 304387.17; 15: 304387.92; 16: 304394.56. Lanes 1–16: *H1* gene detection (57R marker). 1: 300055.32; 2: 300063.9; 3: 300065.4; 4: 300135.14; 5: 300135.3; 6: 300137.31; 7: 301623.15; 8:301624.95; 9: 301026.23; 10: 301037.85; 11: 301041.26; 12: 301044.36; 13: 301045.74; 14: 301055.53; 15: 301056.54; 16: 302498.70.
